# An alkalinization–phytocytokine amplification circuit primes distal immunity in plants

**DOI:** 10.1016/j.xplc.2026.101918

**Published:** 2026-05-19

**Authors:** Yi Zhang, Yingjie Tian, Zhaoxi Lu, Shuguo Hou

**Affiliations:** 1 State Key Laboratory of Wheat Improvement, Peking University Institute of Advanced Agricultural Sciences, Shandong Laboratory of Advanced Agricultural Sciences in Weifang, Weifang, China

## Main text

Plants dynamically fine-tune extracellular pH to coordinate growth, development, and stress responses (Zhu et al., 2026). Two canonical paradigms of this regulation are the acid-growth hypothesis and pathogen-induced apoplastic alkalinization. Auxin signaling activates plasma membrane-localized proton pumps to promote proton efflux, thereby acidifying the apoplast to approximately pH 4.5–5.5; this acidic environment promotes cell wall loosening and facilitates cell expansion. In contrast, apoplastic alkalinization has long been recognized as a hallmark of plant immune activation. Since the 1990s, multiple pathogen-derived elicitors, such as flagellin, have been shown to trigger rapid extracellular alkalinization in suspension-cultured cells, establishing extracellular alkalinization as a useful bioassay for identifying immunogenic elicitors (Felix et al., 1999). Some plant-derived small peptide signals, such as rapid alkalinization factors, have also been shown to induce apoplastic alkalinization (Pearce et al., 2001). Emerging studies have further revealed that immune-triggered alkalinization (ITA) results from the suppression of plasma membrane H^+^-ATPase activity (Elmore and Coaker, 2011). However, the underlying biochemical mechanisms, particularly the biological significance of apoplastic pH elevation during immunity, have remained largely elusive. A recent study (Wang et al., 2026) addresses these questions by linking immune activation, apoplastic pH dynamics, and pathogen resistance.

Plant immunity comprises two interconnected layers: pattern-triggered immunity (PTI) and effector-triggered immunity (ETI) (Zhou and Zhang, 2020). PTI is activated when plasma membrane-localized pattern recognition receptors perceive pathogen-associated molecular patterns, such as the flagellin epitope flg22. In contrast, ETI is initiated when cytoplasmic nucleotide-binding leucine-rich repeat receptors, such as ZAR1 and RPS4, detect specific effector proteins, including the *Pseudomonas syringae* pv. *tomato* (*Pst*) effectors HopZ1a and AvrRps4. Using a pH-sensitive fluorescent dye, Wang et al. (2026) monitored dynamic changes in apoplastic pH in *Arabidopsis* leaves during PAMP treatment and pathogen infection. They found that flg22 treatment, infection with *Pst* D36E, an effector-deficient derivative of *Pst* DC3000 that specifically triggers PTI, and infection with *Pst* D36E expressing HopZ1a or AvrRps4 induced a two- to three-unit increase in apoplastic pH. Notably, PTI- and ETI-induced alkalinization mutually potentiated each other, consistent with other immune outputs, yet remained susceptible to suppression by certain effectors. The authors further showed that artificially elevating apoplastic pH with buffer treatment significantly enhanced *Arabidopsis* resistance to pathogens. Together, these findings suggest that ITA contributes directly to plant disease resistance (Wang et al., 2026).

**Figure 1:**
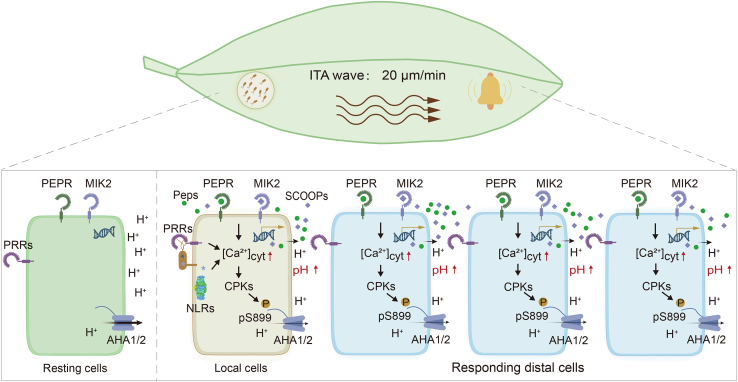
Working model of ITA propagation and its role in immune priming. Pattern-triggered immunity (PTI) and effector-triggered immunity (ETI) induce apoplastic alkalinization, termed immune-triggered alkalinization (ITA), through calcium-dependent protein kinase (CPK)-mediated phosphorylation of the H^+^-ATPase AHA1 at Ser899, which suppresses its proton-pumping activity. Concurrently, immune-activated cells secrete mobile phytocytokines, including plant elicitor peptides (Peps) and serine-rich endogenous peptides (SCOOPs), which are perceived by PEP RECEPTORs (PEPRs) and MALE DISCOVERER1-INTERACTING RECEPTOR-LIKE KINASE 2 (MIK2), respectively, thereby triggering apoplastic alkalinization and inducing the expression of the corresponding phytocytokine genes. Apoplastic alkalinization sensitizes plant responses to these phytocytokines. Together, apoplastic alkalinization and phytocytokine signaling establish an amplification circuit that sustains ITA propagation and promotes immune priming in distal tissues. PRRs, plasma membrane-localized pattern recognition receptors; NLRs, nucleotide-binding leucine-rich repeat receptors; [Ca^2+^]cyt, cytosolic calcium transients.

The *Arabidopsis* autoinhibited plasma membrane H^+^-ATPases AHA1 and AHA2 are key regulators of apoplastic alkalinization. Accumulating evidence indicates that AHA activity is tightly controlled by phosphorylation at specific residues, and phosphorylation at Ser899 may suppress AHA1/2 activity during ITA (Nuhse et al., 2007). The authors developed a phospho-specific antibody against AHA1 Ser899 and detected a marked increase in Ser899 phosphorylation following flg22 treatment and bacterial infection. Moreover, by generating phospho-mimetic S899D and phospho-dead S899A transgenic *Arabidopsis* lines in the *aha1*/*aha2* background, they provided compelling evidence that Ser899 phosphorylation is required for ITA and contributes to resistance against bacterial pathogens. The authors further showed that calcium-dependent protein kinases (CPKs) phosphorylate AHA1 at Ser899, as this residue lies within a conserved CPK recognition motif. Genetic and biochemical analyses demonstrated that clade II CPKs, including CPK3, CPK9, CPK29, and CPK33, redundantly mediate flg22- and pathogen-induced phosphorylation of AHA1 at Ser899, thereby attenuating its activity and promoting apoplastic alkalinization (Wang et al., 2026) (Figure 1).

Interestingly, the authors showed that ITA propagates from infected cells into neighboring uninfected cells. The occurrence of distal apoplastic alkalinization in the absence of pathogen spread suggests the involvement of a systemic immune signaling process rather than a direct pathogen-triggered response. N-hydroxypipecolic acid, reactive oxygen species (ROS), and Ca^2+^ are well-established mobile signals that coordinate systemic acquired resistance and wounding responses in plants (Toyota et al., 2018; Sun and Zhang, 2021). However, distal ITA persists in the key signaling mutants *ald1*, *rbohD*, and *glr3.3*/*glr3.6*, suggesting that this process is independent of these canonical pathways. Notably, the propagation speed of ITA, approximately 20 μm/min, is much slower than that of previously described systemic responses, further implying that distal ITA operates through a distinct immune signaling mechanism. Importantly, distal ITA still requires pathogen-induced phosphorylation of AHA1/2. Although the apoplast is an open extracellular space, distal ITA does not occur via passive diffusion, as application of an H^+^-ATPase inhibitor at the transition zone blocks distal alkalinization (Wang et al., 2026). These results indicate that alkalinization is actively re-established in adjacent uninfected cells. The authors further showed that local ITA not only directly activates distal immune responses, including MAPK activation and the expression of alkalinization-primed genes, but also enhances pathogen-induced immune activation in distal tissues. Together, these findings support a model in which ITA orchestrates both immediate immune activation and long-term immune priming in distal cells. Notably, blocking alkalinization propagation suppresses distal immunity, whereas alkalinization alone is insufficient to directly trigger immune responses (Wang et al., 2026). This pattern suggests the existence of an unknown signal that acts synergistically with alkalinization to regulate distal immunity.

Pathogen-challenged plant cells can produce immunomodulatory peptides known as phytocytokines (Hou et al., 2021). Among them, plant elicitor peptides (Peps) and serine-rich endogenous peptides (SCOOPs) are recognized by their cognate receptors, PEP RECEPTOR1/2 (PEPR1/2) and MALE DISCOVERER1-INTERACTING RECEPTOR-LIKE KINASE 2 (MIK2), respectively, thereby activating immune signaling. Among the genes primed by alkalinization, the authors identified multiple *Pep* and *SCOOP* genes, which encode PROPEP and PROSCOOP precursor peptides, respectively. They also detected elevated Pep3 levels at distal sites following local pathogen infection. Importantly, simultaneous disruption of both peptide pathways significantly suppresses distal ITA, supporting the idea that Peps and SCOOPs function as mobile signals during this process. Although PROPEPs and PROSCOOPs show very low basal expression, they are strongly induced by their cognate peptides, indicating a self-amplifying property. Notably, low-dose Pep1 or SCOOP4 treatment (1 nM) induces PROPEP expression at pH 6.5, but not at pH 5.5, indicating that ITA sensitizes plant cells to these phytocytokines. In turn, both classes of phytocytokines promote extracellular alkalinization, thereby sustaining the propagation of a coupled alkalinization–phytocytokine positive-feedback loop across neighboring cells (Wang et al., 2026) (Figure 1).

This study reveals a novel disease resistance mechanism mediated by extracellular alkalinization. In the proposed model, pathogen-induced increases in apoplastic pH prime plants for heightened local and systemic immune activation upon pathogen challenge. The authors propose that this sensitization may result from strengthened interactions between immune ligands and their receptors. Consistent with this idea, Pep binding to PEP RECEPTOR 1 is enhanced under alkaline conditions (Liu et al., 2022). In addition, membrane depolarization has been shown to modulate the activity of the NADPH oxidase that mediates ROS production, as well as that of plasma membrane-resident ion channels, including calcium and anion channels (Zhu et al., 2026). Whether alkalinization regulates immunity through additional mechanisms, such as crosstalk with ROS and Ca^2+^ signaling, remains to be determined. The study demonstrates that alkalinization confers resistance to multiple pathogens, including the bacterium *P. syringae* and the fungus *Fusarium oxysporum*. However, whether this mechanism confers broad-spectrum disease resistance in plants remains unclear. Notably, apoplastic acidification has also been reported to potentiate defense responses in certain incompatible plant–microbe interactions (Elmore and Coaker, 2011), whereas *F. oxysporum* secretes rapid alkalinization factor-like effectors to induce apoplastic alkalinization and promote virulence (Masachis et al., 2016). These findings point to a complex interplay between apoplastic pH and pathogen resistance, most likely influenced by both pathogen lifestyle and the temporal progression of infection. Future studies integrating diverse plant–pathogen systems with spatiotemporal omics and high-resolution imaging will help resolve these questions. Such work will also provide an important theoretical foundation for developing broad-spectrum disease resistance in crops through gene editing, optimized cultivation, and inducible defense strategies.

Establishing distal immunity during local infection is a crucial defense strategy for limiting pathogen invasion and spread. This study reveals a novel signaling pathway that controls distal immunity through a mechanism distinct from known systemic immune signaling pathways. Specifically, this pathway mediates local-to-distal communication within leaves, triggering rapid immune activation while establishing longer-term resistance priming in distal tissues. Such resistance priming contributes to protection against subsequent pathogen challenge. Previous studies have shown that a strong local defense response, termed localized acquired resistance (Jacob et al., 2023), is activated in cells surrounding infection sites and likely functions to prevent pathogen spread. It has therefore been proposed that mobile signals originating from infected tissues establish immune priming in adjacent cells to restrict pathogen dissemination. Whether the alkalinization–phytocytokine circuit contributes to this process remains to be investigated. In addition, the duration of alkalinization–phytocytokine-mediated distal responses, as well as their potential for long-distance transmission to distant leaves, warrant further study.

Recent breakthroughs have firmly established phytocytokines as central regulators of plant immunity. Although their autocrine-like roles in local immune regulation have been extensively characterized, their potential functions as paracrine- or endocrine-like signals in orchestrating systemic immunity remain poorly understood. This study presents a new signaling paradigm in which phytocytokines coordinate with ITA to establish local–distal communication networks, and it provides direct experimental evidence supporting a paracrine mode of phytocytokine signaling in immune regulation. These findings raise several important mechanistic questions. First, plants produce a repertoire of phytocytokines that may trigger apoplastic alkalinization, but only SCOOP and Pep have been shown to regulate the ITA wave in *Arabidopsis*. Does a similar mechanism apply to other phytocytokine families outside Brassicaceae? Second, beyond apoplastic alkalinization, are there additional molecular determinants that facilitate phytocytokine function as systemic immune signals? Third, can phytocytokines move through symplastic routes via plasmodesmata, in addition to apoplastic transport? Finally, by analogy with animal cytokines, do plants possess specialized immune-active cell types or states that produce distinct phytocytokines, and if so, how do they communicate with surrounding cells? Addressing these questions will bridge critical gaps in our understanding of how phytocytokines coordinate plant immunity.

## Funding

This work was supported by grants from the Taishan Scholars Program (tsqn202507313), the Shandong Provincial Key R&D Program of China (2025CXPT175), the National Key R&D Program of China (2022YFF1001700), the Yuandu Scholars Program (ydxz2024014), and the National Natural Science Foundation of China (32472567).

## Acknowledgments

We apologize to the authors whose valuable work could not be cited in this commentary because of space constraints. No conflict of interest declared.

## Author contributions

Y.Z., Y.T., Z.L., and S.H. wrote the manuscript, and Y.Z. and Y.T. prepared the schematic figure. All authors read and approved the final manuscript.
